# Risky sexual behavior and associated factors among reproductive age women in eastern African countries: a multilevel analysis of the recent demographic and health survey

**DOI:** 10.1186/s12905-024-03107-x

**Published:** 2024-04-30

**Authors:** Belayneh Shetie Workneh, Alebachew Ferede Zegeye, Tadesse Tarik Tamir, Enyew Getaneh Mekonen

**Affiliations:** 1 Department of Emergency and Critical Care Nursing, School of Nursing, College of Medicine and Health Sciences, University of Gondar, Gondar, Ethiopia; 2 Department of Medical Nursing, School of Nursing, College of Medicine and Health Sciences, University of Gondar, Gondar, Ethiopia; 3 Department of Pediatrics and Child Health Nursing, School of Nursing, College of Medicine and Health Sciences, University of Gondar, Gondar, Ethiopia; 4 Department of Surgical Nursing, School of Nursing, College of Medicine and Health Sciences, University of Gondar, Gondar, Ethiopia

**Keywords:** Women, Sexual behavior, Eastern Africa, Risky sex

## Abstract

**Introduction:**

Risky sexual behavior exposes an individual to the risk of contracting sexually transmitted infections including human immunodeficiency virus (HIV). Even though risky sexual behavior is a devastating problem in low- and middle-income countries, studies on risky sexual behavior and associated factors among reproductive-age women in Eastern African countries are limited. Therefore, this study aimed to assess the magnitude of risky sexual behavior and associated factors among reproductive-age women in Eastern African countries that help to target high-risk groups and set appropriate intervention.

**Method:**

The appended and recent Demographic and Health Survey dataset of 10 Eastern African countries from 2012 to 2022 was used for data analysis. A total of 111,895 participants were included in this study as a weighted sample. Associated factors were determined using a multilevel mixed-effects logistic regression model. Significant factors in the multilevel mixed-effect logistic regression model were declared significant at p-values < 0.05. The adjusted odds ratio (AOR) and confidence interval (CI) were used to interpret the results.

**Result:**

The overall magnitude of risky sexual behavior among reproductive-age women in Eastern African countries was 28.16% (95% CI 27.90%, 28.43%), which ranged from 3.80% in Ethiopia to 67.13% in Kenya. In the multivariable analysis, being a younger woman, being an educated woman, being tested for human immunodeficiency virus, having work, drinking alcohol, and being an urban dweller were factors that were significantly associated with higher odds of risky sexual behavior.

**Conclusion:**

The overall magnitude of risky sexual behavior among reproductive-age women in Eastern African countries was high. Individual-level (being a younger woman, being an educated woman, being tested for human immunodeficiency virus, having work, and drinking alcohol) and community-level (being an urban dweller) variables were associated with higher odds of risky sexual behavior. Therefore, policymakers and other stakeholders should give special consideration to urban dwellers, educated, worker and younger women. Better to improve the healthy behavior of women by minimizing alcohol consumption and strengthening HIV testing and counseling services to reduce the magnitude of risky sexual behavior.

## Introduction

Risky sexual behavior is defined as sexual activities that expose an individual to the risk of having sexual and reproductive health problems [1]. It includes engaging in unprotected sexual behavior, having sex at a young age, having sex with multiple sexual partners, and having sex while under the influence of drugs or alcohol. The risk of acquiring a sexually transmitted infection (STI) is elevated in individuals with RSB. Adverse outcomes such as infertility and ectopic pregnancies are also linked to STIs. Cervical cancer is also brought on by the human papillomavirus, which also causes genital warts. Similarly, the chance of HIV transmission rises due to the presence of STIs. To prevent the harmful effects of unsafe sex (HIV, unintended pregnancy, incurable STI, etc.), creating awareness of responsible sex behavior is critical [2].

Despite the social, economic, demographic, and health benefits of safe sex and consistent condom use is important in promoting women’s health and well-being, the findings of the previous studies highlighted that, the prevalence of risky sexual behavior, 17.2–24.7% in Ethiopia [3, 4], 66.9% in Ghana [5], 28.7% in Rwanda [6], 69.5% in Bangkok [2]. The prior studies revealed that age, educational status, working status, age at first sexual intercourse, drinking alcohol, wealth index, marital status, residence, comprehensive knowledge about HIV/ADIS, tested for HIV, and ever heard about sexually transmitted infections (STI) were factors significantly associated with risky sexual behavior [2–4, 6–12].

Due to the negative consequences of risky sexual behavior on women’s health, there must be an intervention to reduce the magnitude of risky sexual behavior and the spread of sexually transmitted infections. Even though risky sexual behavior is a devastating problem in low- and middle-income countries, studies on risky sexual behavior and associated factors among reproductive-age women in Eastern African countries are limited. Therefore, this study aimed to assess the magnitude of risky sexual behavior and associated factors among reproductive-age women in Eastern African countries that help to target high-risk groups and set appropriate interventions.

## Methods

### Data source, study setting, period, and design

The study utilized the appended and the recent demographic and health survey dataset from 10 Eastern African countries conducted from 2012 to 2022. DHS is a community-based cross-sectional study conducted every five years to examine health and health-related indicators.

### Study population, sampling technique, and analysis

The recent DHS data of 10 Eastern African countries (Ethiopia, Comoros, Burundi, Kenya, Mali, Nigeria, Rwanda, Zimbabwe, Tanzania, and Zambia) was downloaded from the Demographic Health Survey (DHS) program website and appended to have a single data set. DHS data exhibit nested dependencies, where individuals are nested within communities. It employs stratified two-stage cluster sampling. Clusters (communities) are sampled, and within each cluster, households and individuals are further selected. The cleaned and recorded data were analyzed using STATA (version 14) statistical software. The women’s sample weight (v005/1,000,000) was applied to address under or over-sampling. Variance inflation factor (VIF) was tested to check multi-collinearity. To determine factors associated with the outcome variable (risky sexual behavior) multi-level mixed-effect logistic regression was applied. We conducted a likelihood ratio test, comparing ordinary logistic regression to multilevel logistic regression. The results affirmed a significant improvement when using multilevel models, reinforcing their suitability. Multi-level mixed-effect logistic regression follows four models: null model (outcome variable only), model I (only individual-level variables), model II (only community-level variables), and model III (both individual and community-level variables). The model without independent variables (Null model) was used to check the variability of risky sexual behavior across the cluster. The association of individual-level variables with the outcome variable (Model I) and the association of community-level variables with the outcome variable (Model II) were assessed. In the final model (Model III), the association of both individual and community variables was fitted with the outcome variable (risky sexual behavior). Variables with a p-value of < 0.25, were candidates for the multivariable analysis in univariate analysis at 95% confidence intervals and variables with a p-value of ≤ 0.05 were considered as significantly associated with the outcome variable in the final analysis. The weighted total sample participants for the study were 111,895 (Table 1).

**Table 1:** Sample size for risky sexual behavior among reproductive-age women in Eastern African countries

Country	Year of survey	Weighted sample(*n*)	Weighted sample (%)
Burundi	2016/17	10,224	9.14
Comoros	2012	3,261	2.91
Ethiopia	2016	10,301	9.21
Kenya	2022	27,703	24.76
Mali	2018	8,600	7.69
Rwanda	2019/20	8,613	7.7
Tanzania	2022	12,011	10.73
Uganda	2016	13,682	12.23
Zambia	2018	10,297	9.2
Zimbabwe	2015	7,204	6.44
Total sample size		111,895	

### Study variables

#### Dependent variable

The dependent variable of this study was risky sexual behavior among reproductive-age women derived from a series of questions from the individual records. The woman was considered to have risky sexual behavior if she experienced either or both of the risky sexual behaviors, (I) had multiple sexual partners, and (II) had sexual intercourse other than a spouse or live-in partner. Even though Condon’s use during sex is one of the most effective methods to protect oneself from sexually transmitted infections including HIV [13], the authors of this study didn’t include it to determine the outcome. Because the DHS data incorporate condom use in the last sexual intercourse only. Thus, the consistency of condom utilization during sexual intercourse is unknown.

#### Independent variables

The independent variables of this study were individual-level variables, age (15–19 years, 20–35 years, and 36–49 years), educational level (no education, primary, secondary, and higher level), having media exposure (yes or no), household wealth index (poor, middle and rich), working status (working or not working), tested for HIV (yes, no), drinking alcohol (yes or no) have comprehensive knowledge about HIV/ADIS), age at first sex (< 18 years or ≥ 18 years), and community level variables, residence (urban or rural), community level media exposure (low or high), community level women illiteracy (low or high), community level poverty (low or high). The community media exposure, community poverty, and community illiteracy levels were aggregated from the individual level variables; media exposure (derived from combining whether a respondent reads a newspaper, watches television, and listens to radio and coded as yes (if the respondent had been exposed to at least for one of these media) an no (otherwise), house wealth status (wealth index), and maternal educational status. Regarding the analysis of the aggregation, first, the individual variables were re-categorized and cross-tabulations were done with the cluster variable using STATA version 14. Then, the proportion of media exposure, poverty, and illiteracy was computed using Microsoft Excel 2013. Next, the proportions from Excel were imported to STATA and combined with the original set of the variables in the STATA. Finally, we have categorized the proportion of media exposure, poverty, and illiteracy into levels.

## Result

### Individual and community-level characteristics of the study participants

More than half (60.49%) of the participants were in the age category of 20–35 years. More than two-thirds of (72.51%) respondents had media exposure. More than half (60.21%) of the participants in the study had work. The majority (78.99%) of the respondents were tested for HIV. The majority (95.36%) of women’s ever heard about STIs. More than two-thirds (69.68%) of the participants were rural dwellers (Table 2).

**Table 2:** Individual and community-level characteristics of the study participants

Individual level variables		Weighted frequency (*n*)	Percentage (%)
Age	15–19 years	11,042	9.87
	20–35 years	67,686	60.49
	36–49 years	33,166	29.64
Educational level	No education	25,363	22.67
	Primary	46,480	41.54
	Secondary	30,638	27.38
	Higher	9414	8.41
Marital status	Single	14,271	12.75
	Married	72,097	64.43
	Ever married	25,527	22.81
Media exposure	No	30,755	27.49
	Yes	81,140	72.51
Household wealth	Poor	40,670	36.35
	Middle	21,567	19.27
	Rich	49,658	44.38
Currently working	No	44,520	39.79
	Yes	67,375	60.21
Tested for HIV	No	20,268	21.01
	Yes	76,187	78.99
Drinking alcohol	No	13,645	66.48
	Yes	6880	33.52
Know HIV/ADIS	No	65,077	93.64
	Yes	4422	6.36
Ever heard about STI	No	4477	4.64
	Yes	91,978	95.36
Residence	Urban	33,927	30.32
	Rural	77,967	69.68
Community-level media exposure	Low	60,640	54.19
	High	51,255	45.81
Community-level women illiteracy	Low	61,484	54.95
	High	50,411	45.05
Community level poverty	Low	55,100	49.24
	High	56,795	50.76

### Prevalence of risky sexual behavior among reproductive-age women in eastern African countries

**Fig. 1:**
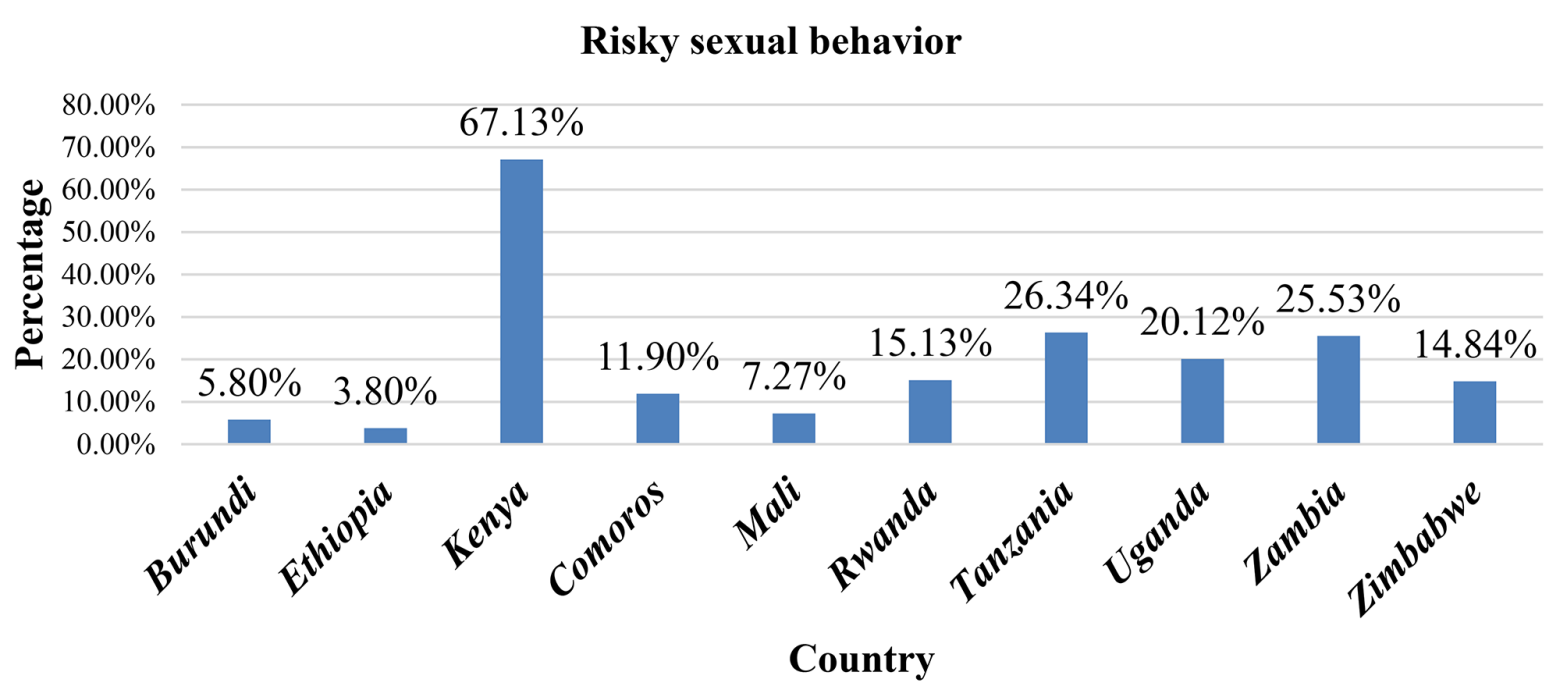
Prevalence of risky sexual behavior among reproductive-age women in Eastern African countries

The overall magnitude of risky sexual behavior among reproductive-age women in Eastern African countries was 28.16% (95% CI 27.90%, 28.43%), which ranged from 3.80% in Ethiopia to 67.13% in Kenya (Fig. 1).

### Types of risky sexual behavior among Reproductive-age women in eastern African countries

From the total participants of the study, 17,994 (16.08%), and 30,496 (27.25%) of the women reported that they had two or more sexual partners, and had sexual intercourse other than a spouse or live-in partner (Table 3).

**Table 3:** Types of risky sexual behavior among reproductive-age women in Eastern African countries

Item		Weighted frequency (*n*)	Percentage (%)
Had two or more sexual partners	No	93,901	83.92
	Yes	17,994	16.08
Had sexual intercourse other than a spouse or live-in partner	No	81,398	72.75
	Yes	30,496	27.25

### Multivariable multilevel logistic regression of risky sexual behavior among reproductive-age women in eastern African countries

In the final fitted model of multivariable logistic regression being younger age, being educated, drinking alcohol, not being tested for HIV, being a worker, and being an urban dweller were factors significantly associated with higher odds of risky sexual behavior (Table 4).

The odds of risky sexual behavior were 13.06 (AOR = 13.06, 95% CI: 9.98, 17.09) and 1.81 (AOR = 1.81, 95% CI: 1.48, 2.21) times higher among 15 to 19 years and 20 to 35 years old women as compared with women aged 36 to 49 years respectively. The likelihood of risky sexual behavior was 1.36 (AOR = 1.36, 95% CI: 1.12, 1.65), 3.86 (AOR = 3.86, 95% CI: 3.03, 4.92), and 4.13 (AOR = 4.13, 95% CI: 2.93, 5.83) times higher among women who had primary, secondary, and higher educational level as compared with those women who had no education respectively. Risky sexual behavior was 1.73 (AOR = 1.73, 95% CI: 1.46, 2.06) times higher among worker women as compared with their counterparts. The odds of risky sexual behavior were 1.75 (AOR = 1.75, 95% CI: 1.48, 2.06) times higher among women who drank alcohol as compared with women who didn’t drink alcohol. The likelihood of risky sexual behavior was 1.43 (AOR = 1.43, 95% CI: 1.17, 1.74) times higher among women who didn’t test for HIV as compared with women tested for HIV. Risky sexual behavior was 3.73 (AOR = 3.73, 95% CI: 2.89, 4.80) time higher among urban dwellers as compared with rural dwellers. There was about 29.73% variability in risky sexual behavior due to the difference between communities/clusters. The final model (model III), attributed approximately 55.89% of the variation in the likelihood of risky sexual behavior to both individual and community-level variables. The lowest deviance, which was 6293.009 in model III revealed that model III was the best fit for the data.

**Table 4:** Multivariable multilevel logistic regression analysis of individual-level and community-level factors associated with risky sexual behavior among reproductive-age women in Eastern African countries

Parameter	Null model	Model I AOR (95% CI)	Model II AOR (95% CI)	Model III AOR (95% CI)
Age				
15–19 years		12.14 (9.29, 15.85)		13.06 (9.98, 17.09)*
20–35 years		1.76 (1.44, 2.15)		1.81 (1.48, 2.21)*
36–49 years (ref.)				
Age at first sex				
< 18 years		1.23 (1.05, 1.45)		1.17 (1.00, 1.38)
≥ 18 years (ref.)				
Level of education				
No education (ref.)				
Primary		1.45 (1.20, 1.76)		1.36 (1.12, 1.65)*
Secondary		4.91 (3.88, 6.21)		3.86 (3.03, 4.92)*
Higher		7.11 (5.12, 9.88)		4.13 (2.93, 5.83)*
Currently working				
No (ref.)				
Yes		1.62 (1.36, 1.91)		1.73 (1.46, 2.06)*
Household wealth				
Poor		0.85 (0.70, 1.05)		1.21 (0.96, 1.51)
Middle		0.71 (0.56, 0.90)		1.03 (0.80, 1.33)
Rich (ref.)				
Drink alcohol				
No (ref.)				
Yes		1.63 (1.38, 1.92)		1.75 (1.48, 2.06)*
Knowledge about HIV/ADIS				
No (ref.)				
Yes		1.22 (0.87, 1.71)		1.25 (0.89, 1.76)
Have media exposure				
No (ref.)				
Yes		1.09 (0.92, 1.29)		1.02 (0.86, 1.21)
Tested for HIV				
No (ref.)				
Yes		1.33 (1.09, 1.62)		1.43 (1.17, 1.74)*
Residence				
Rural (ref.)				
Urban			2.16 (2.08, 2.23)	**3.73 (2.89, 4.80)** ^*^
Community-level media exposure				
Low (ref.)				
High			2.60 (2.26, 2.98)	1.01 (0.78, 1.30)
Community-level women illiteracy				
Low (ref.)				
High			2.36 (2.07, 2.71)	1.08 (0.86, 1.36)
Community level poverty				
Low (ref.)				
High			0.63 (0.57, 0.70)	0.94 (0.74, 1.18)
Variance	1.392	0.605	0.849	0.614
ICC	29.73	15.53	20.51	15.73
PCV	Reference	56.54	39.01	55.89
Deviance	127,666.856	6405.148	125,188.016	6293.009

AOR, adjusted odds ratio; ICC, intra-class correlation coefficient; PCV, proportional change in variance; (ref.), reference; ^*^p ≤ 0.05 (significantly associated)

## Discussion

This study aimed to assess risky sexual behavior and associated factors among reproductive-age women in Eastern African countries. The overall magnitude of risky sexual behavior among reproductive-age women in Eastern African countries was 28.16% (95% CI 27.90%, 28.43%), which ranged from 3.80% in Ethiopia to 67.13% in Kenya. It implies that risky sexual behavior is still a great concern in Eastern African countries. Being younger, being educated, having work, drinking alcohol, being tested for HIV, and being an urban dweller were factors associated with the higher odds of risky sexual behavior.

The odds of risky sexual behavior were higher among women who drink alcohol as compared with women who never drink alcohol. The finding of this study are consistent with the prior studies [14–19]. Alcohol plays a great role in negative behaviors. Excessive drinking can lower inhibitions, impair a person’s judgment, and increase the risk of aggressive behaviors [20]. Drinking alcohol resulted in conflict between an individual’s desire and their inhibitions, which in turn failed to inhibit inappropriate or risky behaviors [21]. It could be because women who drink alcohol may not be fully conscious which in turn alters their decision in whether risky or responsible sexual activity. Furthermore, it could be because women intentionally use alcohol for their sexual interest which in turn results in risky sexual practice.

The current finding revealed that risky sexual behavior was higher among women who had work compared with their counterparts. This finding is supported by the previous study [12, 22]. It might be because women who had work exhibit higher levels of independence and tend to go against certain societal expectations, which in turn can cause having multiple sexual partners and committing sexual intercourse other than their spouse or live-in partner.

The age of women is another significant factor associated with risky sexual behavior. The odds of risky sexual behavior were significantly higher among younger women as compared with older women. This finding is supported by the prior study [12]. Compared to older women, younger women were better educated, made their sexual debuts earlier, and were more likely to engage in high-risk and transactional sex [11]. Furthermore, it could be due to variations in sexual desire between younger and older women. Compared to older women, younger women experienced more frequent and intense sexual desires. Additionally, they were more likely to have sex earlier in a relationship and had more sex overall [23].

The odds of risky sexual behavior were higher among women who were educated as compared with those women who had no education. It could be due to variations in their residence and age between educated and non-educated women. There is a significant education gap between urban and rural areas [24]. However, additional investigation is required to gain a deeper comprehension of the complex correlation between education and risky sexual behaviors among reproductive-age women.

Contrary to the prior study [25], in this study, the likelihood of risky sexual behavior was higher among women not tested for HIV as compared with women tested for HIV. It could be because those women who have tested for HIV have a chance of getting information from health professionals regarding the negative effects of risky sexual behavior and the burden of sexually transmitted infections. Thus, women tested for HIV may not participate in risky sexual activities.

In addition, contrary to the prior studies [26, 27], the odds of risky sexual behavior were higher among urban dweller women as compared to rural dwellers. It could be because, urban women frequently use social media more likely to share sexually explicit texts, images, and videos with a sexual partner, which in turn leads to risky sexual behavior.

### Strengths and limitations of the study

The data used for the study was nationally representative with multi-country and appropriate statistical analysis which is multilevel analysis. Hence policymakers and the international community can use it as evidence to undertake necessary measures. However, the study also has limitations, important factors that could have a big impact on risky sexual behavior, like beliefs, risky sexual behavior, and social norms, are not included in the dataset. Additionally, due to the sensitive nature of risky sexual behavior, a social desirability bias and under-report may have been present in the women’s verbal responses. These will hinder our findings from having the intended impact, so further studies should be carried out to explore women’s risky sexual behavior.

## Conclusion

The overall magnitude of risky sexual behavior among reproductive-age women in Eastern African countries was high. Individual-level (being a younger woman, being an educated woman, being tested for human immunodeficiency virus, having work, and drinking alcohol) and community-level (being an urban dweller) variables were associated with higher odds of risky sexual behavior. Therefore, policymakers and other stakeholders should give special consideration to urban dwellers, educated, worker and younger women. Better to improve the healthy behavior of women by minimizing alcohol consumption and strengthening HIV testing and counseling services to reduce the magnitude of risky sexual behavior. Furthermore, the authors of the study recommend further investigation into the reason why being educated and working women increases the risk of having risky sexual behavior.

## Data Availability

The datasets generated and/or analyzed during the current study are available publicly online at (https://www.dhsprogram.com).
